# Sex-Specific Association of Low Muscle Mass with Depression Status in Asymptomatic Adults: A Population-Based Study

**DOI:** 10.3390/brainsci14111093

**Published:** 2024-10-30

**Authors:** Sung Joon Cho, Sra Jung, Mi-Yeon Lee, Chul Hyun Park

**Affiliations:** 1 Department of Psychiatry, Kangbuk Samsung Hospital, Sungkyunkwan University School of Medicine, Seoul 03181, Republic of Korea; sungjoon.cho@samsung.com; 2 Workplace Mental Health Institute, Kangbuk Samsung Hospital, Seoul 04514, Republic of Korea; 3 Department of Psychiatry, CHA University Ilsan CHA Hospital, Goyang-si 10414, Republic of Korea; srajung890@chamc.co.kr; 4 Division of Biostatistics, Department of R&D Management, Kangbuk Samsung Hospital, Sungkyunkwan University School of Medicine, Seoul 03181, Republic of Korea; my7713.lee@samsung.com; 5 Department of Physical and Rehabilitation Medicine, Kangbuk Samsung Hospital, Sungkyunkwan University School of Medicine, Seoul 03181, Republic of Korea

**Keywords:** muscular atrophy, sarcopenia, depression, sex differences

## Abstract

Background: The objective of this study was to examine the correlation between low muscle mass (LMM) and depression, with a specific focus on identifying the sex-specific relationship between LMM and depression in a large sample. Methods: This population-based cross-sectional study involved 292,922 community-dwelling adults from 2012 to 2019. Measurements were taken using the Center for Epidemiological Studies Depression (CESD) scale and body composition analyses. Depression was defined as a CESD score ≥ 16, and severe depression as a CESD score ≥ 22. LMM was defined as an appendicular muscle mass/height^2^ below 7.0 kg/m^2^ in men and below 5.4 kg/m^2^ in women. Sex-based multivariable logistic regression analyzed the LMM–depression association, adjusting for confounders, with depression status and severe depression status as dependent variables. Results: Both men and women in the LMM group had an increased odds of depression (men, adjusted odds ratio = 1.13 [95% confidence interval = 1.03–1.12]; women, 1.07 [1.03–1.23]) and severe depression (men, 1.20 [1.05–1.36]; women, 1.10 [1.04–1.15]) compared to those in the control group. Men showed a stronger association between LMM and the presence of depression (*p* for interaction = 0.025) and the presence of severe depression (*p* for interaction = 0.025) compared to women. Conclusions: Decreased muscle mass was independently associated with increased chances of depression and severe depression in both sexes, with a significantly stronger association in men compared to women. This highlights the potential significance of LMM as a predictor of depression, particularly in men.

## 1. Introduction

Sarcopenia is a progressive and generalized skeletal muscle disorder including the accelerated loss of skeletal muscle mass and function. The term sarcopenia, which means “poverty of flesh” in Greek, was initially defined in the 1980s as an age-related decline in lean muscle mass [1]. Since 2010, the term has evolved to encompass not only muscle mass, but also muscle strength, muscle power, and physical performance levels, incorporating the broader concept of muscle function [2]. In 2016, it was recognized as an independent condition and included as an International Classification of Diseases-10 code [3]. Nevertheless, the most significant characteristic of sarcopenia, consistently observed over a prolonged period, is low muscle mass (LMM). 

Recent studies have demonstrated an association between LMM and depression. In a systematic review and meta-analysis study [4], individuals with sarcopenia, which includes LMM, had a higher prevalence of depression and a positive correlation with depressive symptoms. Depression may lead to a decrease in physical activity, which can cause LMM, and LMM may, in turn, influence depression, suggesting a bidirectional relationship [5,6,7]. Numerous studies have demonstrated that the risk factors for depression include dysregulation of energy metabolism [8], chronic neuroinflammation, and neurotransmitter efficiency [9]. Additionally, previous research has shown that among the therapeutic solutions for depression, exercise plays a crucial role in mitigating depressive neuropathology [10], with physical activity known to influence muscle mass and mental health [11]. Exercise boosts the secretion of myokines like Irisin from skeletal muscles, which play roles in the endocrine and autocrine systems [12]. Irisin has multiple beneficial functions, such as promoting fat browning, increasing energy expenditure, reducing inflammation, and improving mitochondrial function [13]. It also activates pathways related to energy metabolism [14] and memory formation [15], both implicated in depression. This evidence suggests that while depression may exacerbate LMM, there is sufficient evidence to consider LMM as a predictor of depression. A recent study [16] has shown that muscle strength, particularly handgrip strength, is closely associated with successful aging. Successful aging refers to maintaining physical and mental health without major diseases, while also preserving physical and cognitive function [17]. Older adults with higher handgrip strength have been found to be more likely to experience successful aging, indicating that muscle strength decline is a significant risk factor during the aging process. In this context, our study aims to explore the relationship between decreased muscle mass and depression by sex, highlighting the importance of maintaining muscle strength to ensure healthy aging.

Although consistent associations between LMM and depression prevalence have been found in studies conducted worldwide, results in Korean populations have been inconsistent. While studies on older Koreans showed an association between LMM and depression [18,19], a larger study on adults aged 20 and above found no significant association [20]. This discrepancy in findings suggests that additional factors, such as sex differences, may play a role in the relationship between LMM and depression in Korean populations. Both LMM and depression exhibit significant differences in prevalence between sexes, along with sex itself being considered a risk factor for these conditions. Generally, men have a relatively lower prevalence of LMM compared to women, which may be attributed to the fact that men typically have a higher muscle mass. Additionally, with age, women tend to have a lower muscle mass compared to men, which may aggravate muscle strength decline [21]. Furthermore, higher testosterone levels in men can influence their higher muscle mass and strength compared to women [22]. These physiological differences could explain why men and women experience different degrees of risk for LMM and depression.

Depression prevalence is known to be higher in women, with specific factors contributing to this disparity. Women experience hormonal fluctuations, particularly related to estrogen and progesterone, which can influence mood and emotional well-being [23]. Transitions such as pregnancy, childbirth, menstrual cycles, and menopause can be accompanied by hormonal changes that may increase the risk of developing depression [24]. Women often face social and cultural pressures and roles that can contribute to stress and conflict. Additionally, psychological factors, including stress, trauma, and emotional difficulties, can contribute to the development of depression in women [25]. 

Despite notable sex differences and the influence of sex hormones on both LMM and depression, research examining their relationship by sex remains limited. This highlights the necessity of considering sex-specific factors in investigating this link. A nuanced analysis that addresses these differences could clarify the inconsistent findings in Korean populations and enhance our understanding of the underlying mechanisms. Additionally, these inconsistencies present an opportunity to explore demographic, cultural, and healthcare-related factors that may affect the LMM–depression relationship. 

Therefore, this study aims to investigate the sex-specific association of decreased skeletal muscle mass with depression status in a larger sample of the entire adult Korean population. Our hypotheses are as follows:

- Korean adults with LMM may have a higher prevalence of depression in both sexes.
- The negative impact of LMM on the prevalence of depression can differ between men and women.

## 2. Materials and Methods

### 2.1. Study Participants

**Figure 1:**
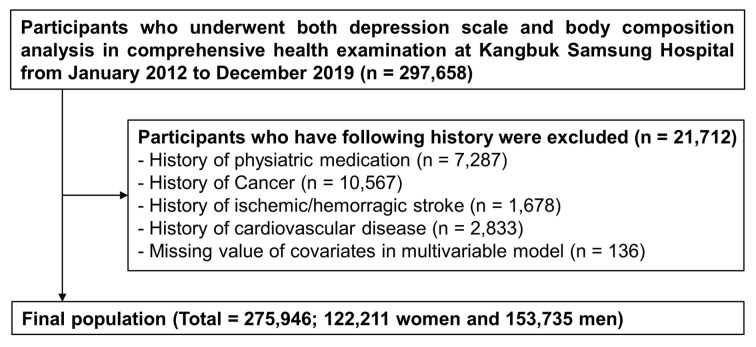
Selection of Study Population.

This two-center, cross-sectional study investigated the association of LMM with the depression status in a Korean population. Participants were recruited from a medical health screening program at the two health promotion centers of the Kangbuk Samsung Hospital, Sungkyungkwan University School of Medicine in Seoul and Suwon, South Korea. A total of 297,658 participants aged 18–95 years underwent depression scale and body composition analysis between 1 January 2012, and 31 December 2019, and were included in the study. Asymptomatic participants were defined as those without the history of psychiatric medication use, any cancer, stroke, or cardiovascular disease. Participants were excluded as follows: a history of psychiatric medication use (*n* = 7287), cancer (*n* = 10,567), stroke and/or were currently using medication for stroke (*n* = 1678), cardiovascular disease (*n* = 2833), or if they had missing covariate values in the multivariable model (*n* = 136) (Figure 1). Finally, a total of 275,946 participants (122,211 women; 153,735 men) were analyzed to determine the association between LMM and depression status. 

This study protocol was approved by the Institutional Review Board (IRB) of our hospital (IRB No. KBSMC 2022-07-017). The requirement for informed consent was exempted by the IRB because we only accessed de-identified datasets routinely collected as part of the health screening exam.

### 2.2. Measurements

Physicians used standardized questionnaires to examine baseline characteristics such as health associated behavior variables (smoking status, alcohol drinking history, and regular physical activity), and medical history (history of diabetes mellitus, hypertension, dyslipidemia, cancer, stroke, and cardiovascular disease). Participants with smoking history were categorized under never, former, or current smoker. Those with alcohol consumption above 20 g/day were included in a heavy drinking group [26,27]. Participants who performed vigorous exercise ≥ three times a week for more than 20 min/session were categorized as a regular physical activity group based on the International Physical Activity Questionnaire-Short Form [28].

Blood sampling and anthropometry were performed by trained nurses or medical laboratory technologists. Systolic and diastolic pressure measurements were performed using a standardized sphygmomanometer after resting for five minutes according to the Hypertension Detection and Follow-up Program protocol. History of hypertension was defined as blood pressure ≥ 140/90 mm Hg or presently taking antihypertensive medication according to the criteria specified in the 8th report of the Joint National Committee on prevention, detection, evaluation, and treatment of high blood pressure. The anthropometric data were measured by experienced nurses. The biochemical parameters included fasting glucose, insulin, glycosylated hemoglobin (HbA1c), total cholesterol, low-density lipoprotein cholesterol (LDL), high-density lipoprotein cholesterol (HDL), and triglycerides (Tg). Furthermore, height and weight of each participant were measured twice and then averaged measurement was used. Body mass index (BMI) was estimated as body weight in kilograms divided by height in meters squared (kg/m^2^).

### 2.3. Measurement and Definition of Low Muscle Mass

The appendicular skeletal muscle mass (kg), as the sum of muscle mass of arms and legs, was measured using the bioelectrical impedance analysis (BIA, InBody 720, Biospace, Republic of Korea). The BIA was calibrated every morning prior to the test and validated for reproducibility and accuracy for analysis of the skeletal muscle mass. The Skeletal Muscle Mass Index (SMI) was calculated using the following formula: SMI (kg/m^2^) = appendicular skeletal muscle mass (kg)/height (m)^2^ [29].

The status of low skeletal muscle mass was defined according to the criteria specified by the Asian Working Group for Sarcopenia (SMI of below 5.7 kg/m^2^ in women and below 7.0 kg/m^2^ in men) [30]. Participants below the LMM cutoff level were included in the LMM group, and the rest, in the control group. 

### 2.4. CESD Score and Definition of Depression

The depressive symptoms were assessed using the Korean version of the 20-item Center for Epidemiological Studies Depression (CESD) scale [31]. It is a self-reported questionnaire with responses measured on a 4-point Likert scale ranging from 0 to 3; a higher total score indicates severe depressive symptoms. Traditionally, a CESD score of 16 or higher is used as the cut-off for screening depression [32]. Therefore, in this study, we defined cases with a CESD score of 16 or higher as the presence of depression. Additionally, based on previous studies [33,34] that indicated that a CESD score of 22 or higher is effective in screening for clinically relevant depression, we defined cases with a CESD score of 22 or higher as severe depression.

### 2.5. Statistical Analysis

Baseline characteristics of study groups were compared based on the one-way analysis of variance (ANOVA) for continuous variables and the χ^2^ test for categorical variables. Prevalence of depression based on the CESD score in each group classified according to the LMM status was compared using the χ^2^ test. Adjusted means (SE, standard error) of the CESD values of the control and LMM groups were compared using the analysis of covariance (ANCOVA) after adjustments for possible confounding factors such as age, center, systolic blood pressure (sBP), glucose level, HDL, smoking status, heavy alcohol use, and regular physical activity. To determine the association of the LMM status with depression and severe depression, multiple logistic regression analyses were performed with adjustments for possible confounding variables. Interactions based on sex difference were conducted using the likelihood ratio test comparing models with and without multiplicative interaction terms. The level of statistical significance was set at two-tailed *p*-value < 0.05. All analyses were conducted using the IBM SPSS version 26.0 (IBM Co., New York, NY, USA).

## 3. Results

### 3.1. Baseline Demographic Characteristics

A total of 275,946 participants were included, including 122,211 (44.3%) women and 153,735 men (55.7%). The mean ages were 41.6 ± 9.4 and 43.0 ± 9.2 years, and the mean SMIs were 6.19 ± 0.61 and 8.09 ± 0.66 kg/m^2^ for women and men, respectively (all *p* < 0.001) (Table 1).

We assessed the normality of each variable’s distribution using graphical methods such as histograms and Q-Q plots, as shown in Supplementary Figure S1. Variables with normal distributions are reported as a mean ± standard deviation, while right-skewed variables are summarized using a median and interquartile ranges (IQR). The baseline characteristics of the control and LMM groups based on sex are presented in Table 2. The prevalence of LMM was 20.79% in women and 4.17% in men. All the variables were significantly different between the groups for both women and men (all *p* < 0.001), except for the proportion of heavy alcohol use in women (*p* = 0.105).

### 3.2. Comparisons of Prevalence of Depression and CESD Score

The prevalence of depression based on the CESD score in the control and LMM groups is presented in Table 3. For women, the prevalence of depression was significantly higher in the LMM group (16.0%) than in the control group (15.0%) (*p* < 0.001). Furthermore, the prevalence of severe depression was significantly higher in the LMM group (8.3%) than in the control group (7.5%) (*p* < 0.001). Men in the LMM group had a higher prevalence of depression and severe depression than those in the control group (all *p* < 0.05)

**Figure 2:**
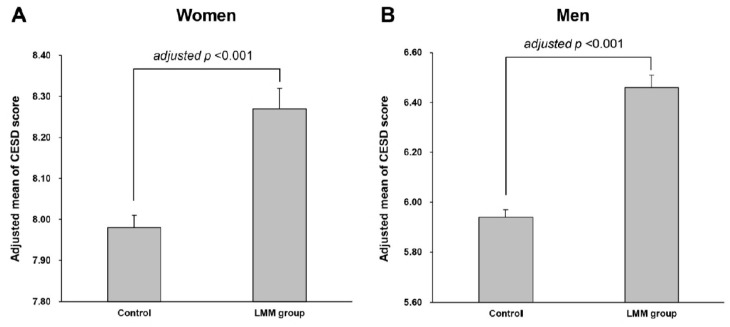
Comparison of adjusted mean CESD scores (with standard errors) of control and LMM groups for (**A**) women and (**B**) men. Adjusted means of CESD scores in groups were estimated using ANCOVA after adjusting for age, center, systolic blood pressure, glucose level, high density lipoprotein cholesterol, smoking status, heavy alcohol use, and regular physical activity. Abbreviations: CESD = center for epidemiological studies depression, LMM = low skeletal muscle mass; ANCOVA = analysis of covariance.

Figure 2 compares the CESD score (as a continuous variable) based on the LMM status for women and men. For women, after adjustments for possible confounding factors such as age, center, sBP, glucose level, HDL-C, smoking status, heavy alcohol use, and regular physical activity, the adjusted mean of the CESD score was significantly higher in the LMM group (adjusted mean [standard error] = 8.27 [0.05]) than that in the control group (adjusted mean [standard error] = 7.98 [0.03]) (adjusted *p* < 0.001). Men in the LMM group (adjusted mean [standard error] = 6.46 [0.08]) had higher adjusted mean of the CESD score than those in the control group (adjusted mean [standard error] = 5.94 [0.02]) (adjusted *p* < 0.001). 

### 3.3. Association Between Depression and Low Skeletal Muscle Mass

Table 4 shows the multivariable logistic regression analyses after adjustments for confounding factors for the association between LMM and the presence of depression and severe depression. For both men and women, in the fully adjusted model, participants with LMM were at an increased odds of having depression than the controls (adjusted odds ratio (OR) [95% CI] for men, 1.13 [1.03–1.12]; for women, 1.07 [1.03–1.12]). The association between LMM and the presence of depression was stronger in men than in women (*p* for interaction = 0.025). 

For severe depression, women and men with LMM were at an increased chance of having depression than the controls (adjusted OR [95% CI] for men, 1.20 [1.05–1.36]; for women 1.10 [1.04–1.15]), showing a stronger association in men than in women (*p* for interaction = 0.028).

## 4. Discussion

This is the first study to explore the sex-specific differences in the relationship between LMM and depression in a large-scale general population of Koreans. The results revealed that LMM was significantly associated with the prevalence of depression and severe depression in both sex groups, even after adjusting for possible confounding factors including regular physical activity. Remarkably, although the prevalence of depression was higher among women compared to men, the association between depression and LMM was stronger in men compared to women.

In this study, men showed significantly high prevalences of diabetes, hypertension and dyslipidemia, high levels in fasting glucose, HbA1c, insulin, total cholesterol, LDL, and TG and low levels in HDL compared to women. This result is consistent with that of a previous study based on the Korean National Health Insurance Service Database from 2009 to 2013 [35]. The higher average age, BMI, and prevalence of unhealthy lifestyle habits such as smoking experience and alcohol abuse history in men could explain the higher incidence of chronic diseases and unhealthy laboratory results despite having higher appendicular skeletal muscle mass, SMI, and regular physical activity compared to women. The proportion of the LMM group was higher in women than in men. Interestingly, while the mean age of the LMM group in women was lower than that of the control group, the mean age of the LMM group in men was higher than that of the control group. This may be due to the social atmosphere in which women want to be skinny and men want to be masculine among young people who are particularly interested in developing an ideal appearance [36,37]. In both sex groups, the prevalence of hypertension and hyperlipidemia was lower and laboratory findings were healthier in the LMM group than in the control group. 

In this study, for both men and women, the severity of depressive symptoms and prevalence of depression and severe depression were significantly higher in the LMM group than in the control group. Even after adjusting for physical activity, the LMM group showed higher rates of depression and severe depression. This suggests that, regardless of physical activity level, LMM is associated with increased depression, supporting LMM as a potential predictive factor for depression. The results of this large-scale study, which includes a relatively young age group, are consistent with those of several previous studies [38,39]. This suggests a significant association between LMM and depression among Koreans. The pathophysiology of LMM has often been associated with increased inflammatory cytokines, which are also intimately associated with depressive symptoms [40]. In particular, IL-6—one important predictor of LMM—is intimately associated with the severity and aggravation of depressive symptoms [41]. Additionally, the dysregulation of energy metabolism, which often accompanies both LMM and depression, can lead to decreased muscle protein synthesis and increased muscle protein breakdown. This metabolic imbalance may further exacerbate muscle loss and depressive symptoms, creating a feedback loop that complicates both conditions [42]. Hormonal imbalances, particularly involving sex hormones such as testosterone and estrogen, can also influence this dynamic. Lower levels of these hormones in aging adults may contribute to a reduced muscle mass while simultaneously increasing their vulnerability to depressive disorders [43,44]. These changes that can occur equally in both sexes may explain the association between LMM and depression observed in both men and women.

Interestingly, the OR of LMM was significantly higher in men than in women in the depression and severe depression groups. In women, the tendency to accumulate adipose tissue is high because of the influence of female hormones [45]. Moreover, because of the lower levels of male hormones in women, the increase in muscle mass is less in women, despite the same level of physical activity as men [46]. Testosterone significantly impacts LMM and depression by promoting satellite cell activation, proliferation, survival, and differentiation, which are crucial to maintain muscles in adults [47]. Additionally, testosterone promotes muscle protein synthesis, improves intracellular amino acid breakdown and recycling, and enhances motor neuron activity [48]. It also promotes the myogenic lineage of pluripotent stem cells and inhibits differentiation into adipocytes through androgen receptor-mediated pathway, thereby reducing body fat mass and increasing fat-free mass [49]. In men, a decreased testosterone level has several characteristics similar to depression, such as decreased libido, energy, and interest, as well as irritability, depressed mood, and weakness [50]. In many studies [51,52], testosterone levels were lower in depressed individuals than in not-depressed individuals. In particular, studies have shown low testosterone levels in men with severe depressive symptoms and treatment-resistant depression [53,54]. The interaction of the hypothalamus–pituitary axis (HP), which affects depressive symptoms by regulating glucocorticoids, and the hypothalamus–pituitary–gonadal axis (HPG), which affects the secretion of testosterone, also impacts the relationship between testosterone and depression. Glucocorticoid secretion induces a depressed mood, while suppressing pulsatile luteinizing hormone secretion and testosterone secretion [55]. Abnormal testosterone secretion also adversely impacts the occurrence of a depressed mood through the HPA and HPG pathways [56]. Differences in social stigma may also have an effect. As women desire a skinny body as their ideal body image, they are more stressed about obesity [37]. As men desire a masculine body as an ideal body image, muscle mass itself may have a greater impact on men compared to women. Additionally, as various psychosocial factors such as pregnancy and childbirth, discriminatory environment, and learned helplessness are known to affect women’s depression [57] more than men’s, the influence of LMM on depression may be mitigated in women.

## 5. Limitations

This study has several limitations. First, the causal relationship cannot be evaluated because of its retrospective nature. Therefore, longitudinal cohort studies are required to elucidate the associations and extend the current findings. Second, the participants of the medical health screening program at two health promotion centers of the university hospital in South Korea were the participants of the study. Therefore, there may be limitations in terms of generalizing the results of this study to a larger population. However, this study could compensate for this shortcoming because it includes a varied sociodemographic population. Moreover, as the mental health data used in the present study were self-reported, there could be a response bias; however, careless responses were excluded during data processing. Future studies with objective measures could clarify more accurate effects of LMM on depression. Furthermore, due to a lack of information on muscle function or physical performance, definite sarcopenia could not be confirmed in this study. However, many clinical studies have defined sarcopenia based solely on LMM [58,59,60,61], and we categorized participants by their LMM status, as it is one component of sarcopenia. Additionally, muscle strength and performance tend to decrease consecutively with decreasing muscle mass [62]. Thus, LMM remains an important parameter for suggesting the preclinical status of sarcopenia. Lastly, the relatively small number of older participants in our study may limit the analysis of age-related trends, suggesting the need for future research with larger elderly populations.

## 6. Conclusions

In summary, we demonstrated the sex-specific associations of LMM with depression based on the CESD scores among asymptomatic Korean adults. For both sexes, LMM was significantly associated with the prevalence of depression and severe depression. It is noteworthy that the LMM–depression association was stronger in men compared to women. Further research is required for targeting therapies to overcome the LMM status and prevent the occurrence of depression in the general population, especially among men.

## Figures and Tables

**Table 1:** Baseline characteristics in women and men participants.

Characteristics	Overall	Women	Men
Number	275,946	122,211 (44.3)	153,735 (55.7)
Age (year)	42.4 ± 9.3	41.6 ± 9.4	43.0 ± 9.2
Screening center (Seoul, %)	107,463 (38.9)	44,937 (36.8)	62,526 (40.7)
Height (cm)	167.68 ± 8.5	160.52 ± 5.32	173.38 ± 5.84
Weight (kg)	67.31 ± 13.51	57.24 ± 9.02	75.31 ± 10.88
BMI (kg/m^2^)	23.78 ± 3.51	22.22 ± 3.34	25.02 ± 3.12
Appendicular Skeletal Muscle Mass (kg)	20.67 ± 4.93	15.99 ± 2.12	24.39 ± 2.97
* SMI (kg/m^2^)	7.25 ± 1.14	6.19 ± 0.61	8.09 ± 0.66
Smoker status			
Never smoker	145,508 (52.73)	107,595 (88.04)	37,913 (24.66)
Former smoker	81,881 (29.67)	9332 (7.64)	72,549 (47.19)
Current smoker	44,595 (16.16)	2230 (1.82)	42,365 (27.56)
Heavy alcohol use	52,333 (18.96)	6727 (5.5)	45,606 (29.67)
Regular Physical Activity	40,662 (14.74)	15,432 (12.63)	25,230 (16.41)
Comorbidities			
Diabetes (%)	9620 (3.49)	2199 (1.8)	7421 (4.83)
Hypertension (%)	29,385 (10.65)	6237 (5.1)	23,148 (15.06)
Hypertension (%)	46,834 (16.97)	11,883 (9.72)	34,951 (22.73)
Laboratory Finding			
sBP	110.4 ± 12.69	104.84 ± 11.9	114.82 ± 11.5
dBP	71.13 ± 9.78	66.82 ± 8.76	74.56 ± 9.18
Fasting glucose	97.93 ± 16.05	94.23 ± 13.13	100.87 ± 17.49
HbA1c	5.53 ± 0.56	5.44 ± 0.48	5.6 ± 0.62
Insulin ^a^	6.4 (4.4–9.2)	5.9 (4.11–8.42)	6.77 (4.6–9.8)
Total Cholesterol	191.34 ± 34.35	187.55 ± 33.09	194.35 ± 35.02
LDL	126.99 ± 33.24	119.74 ± 31.86	132.75 ± 33.19
HDL	59.67 ± 16.1	67.37 ± 15.96	53.55 ± 13.34
Triglycerides ^a^	97 (68–144)	77 (58–106)	120 (84–173)

^a^ Data are presented as mean ± standard deviation, proportions, or median (interquartile range). * SMI (Skeletal muscle mass index) (%) = appendicular skeletal muscle mass (kg)/height (m)^2^. BMI, body mass index; SMI, skeletal muscle mass index; sBP, systolic blood pressure; dBP, diastolic blood pressure; HbA1c, hemoglobin A1c; LDL, low-density lipoprotein cholesterol; HDL, high-density lipoprotein cholesterol.

**Table 2:** Baseline characteristics according to muscle mass status in women and men.

Characteristics	Women		Men	
	Control	LMM Group	Control	LMM Group
Number	96,800 (79.21)	25,411 (20.79)	147,319 (95.83)	6416 (4.17)
Age (year)	42.01 ± 9.25	40.09 ± 9.82	42.86 ± 9.03	46.01 ± 12.12
Screening center (Seoul, %)	35,963 (37.15)	8974 (35.32)	59,976 (40.71)	2550 (39.74)
Height (cm)	161.09 ± 5.29	158.34 ± 4.86	173.58 ± 5.76	168.6 ± 5.57
Weight (kg)	59.44 ± 8.65	48.86 ± 4.2	76.05 ± 10.45	58.36 ± 5.37
BMI (kg/m^2^)	22.93 ± 3.29	19.51 ± 1.75	25.21 ± 3.02	20.56 ± 1.93
Appendicular Skeletal Muscle Mass (kg)	16.62 ± 1.85	13.59 ± 1.12	24.62 ± 2.8	19.16 ± 1.57
* SMI (kg/m^2^)	6.39 ± 0.51	5.41 ± 0.24	8.15 ± 0.6	6.73 ± 0.26
Smoker status				
Never smoker	84,894 (87.7)	22,701 (89.34)	36,147 (24.54)	1766 (27.52)
Former smoker	7629 (7.88)	1703 (6.7)	69,781 (47.37)	2768 (43.14)
Current smoker	1788 (1.85)	442 (1.74)	40,572 (27.54)	1793 (27.95)
Heavy alcohol use	5392 (5.57)	1335 (5.25)	44,022 (29.88)	1584 (24.69)
Regular Physical Activity	13,157 (13.59)	2275 (8.95)	24,483 (16.62)	747 (11.64)
Comorbidities				
Diabetes (%)	1846 (1.91)	353 (1.39)	7039 (4.78)	382 (5.95)
Hypertension (%)	5344 (5.52)	893 (3.51)	22,322 (15.15)	826 (12.87)
Hypertension (%)	9651 (9.97)	2232 (8.78)	33,742 (22.9)	1209 (18.84)
Laboratory Finding				
sBP	105.78 ± 12.03	101.27 ± 10.67	115.04 ± 11.43	109.81 ± 11.87
dBP	67.25 ± 8.87	65.17 ± 8.11	74.65 ± 9.18	72.43 ± 8.98
Fasting glucose	94.79 ± 13.45	92.12 ± 11.57	100.94 ± 17.31	99.28 ± 21.17
HbA1c	5.46 ± 0.49	5.37 ± 0.4	5.6 ± 0.61	5.58 ± 0.74
Insulin ^a^	6.13 (4.3–8.8)	5.24 (3.72–7.2)	6.87 (4.7–9.93)	4.8 (3.3–6.74)
Total Cholesterol	187.67 ± 33.14	187.1 ± 32.9	194.46 ± 35	191.82 ± 35.44
LDL	120.5 ± 31.98	116.88 ± 31.23	132.98 ± 33.13	127.52 ± 34.22
HDL	66.29 ± 15.88	71.51 ± 15.59	53.27 ± 13.17	59.91 ± 15.36
Triglycerides ^a^	79 (59–110)	72 (56–95)	121 (85–174)	98 (73–137.5)

^a^ Data are presented as mean ± standard deviation, proportions, or median (interquartile range). * SMI (Skeletal muscle mass index, %) = appendicular skeletal muscle mass (kg)/height (m^2^). LMM, low skeletal muscle mass; BMI, body mass index; SMI, skeletal muscle mass index; sBP, systolic blood pressure; dBP, diastolic blood pressure; HbA1c, hemoglobin A1c; LDL, low-density lipoprotein cholesterol; HDL, high-density lipoprotein cholesterol.

**Table 3:** Prevalence of depression according to LMM status in women and men.

	Control	LMM Group	*p* Value
Women			
Number of Participants	96,800	25,411	
Prevalence of Participants with CESD score ≥ 16	15.0%	16.0%	<0.001
Prevalence of Participants with CESD score ≥ 22	7.5%	8.3%	<0.001
Men			
Number of Participants	147,319	6416	
Prevalence of Participants with CESD score ≥ 16	8.5%	9.3%	0.030
Prevalence of Participants with CESD score ≥ 22	3.7%	4.3%	0.029

LMM, low skeletal muscle mass; CESD, Center for Epidemiological Studies Depression.

**Table 4:** Multivariate-adjusted * odds ratios (95% CI) for prevalence of depression by CESD score according to LMM status in women and men.

	Control	LMM Group	*p* for Interaction
Depression (CESD ≥ 16) (%)			
Women	1 (reference)	1.07 (1.03–1.12)	0.025
Men	1 (reference)	1.13 (1.03–1.23)	
Severe Depression (CESD ≥ 22) (%)			
Women	1 (reference)	1.10 (1.04–1.15)	0.028
Men	1 (reference)	1.20 (1.05–1.36)	

* Adjustments for age, center, systolic blood pressure, fasting glucose, high-density lipoprotein cholesterol, smoking status, heavy alcohol use, and regular physical activity. CI, confidence interval; CESD, Center for Epidemiological Studies Depression; LMM, low skeletal muscle mass.

## Data Availability

The data that would be necessary to interpret, replicate, and build upon the methods or findings reported in this article are available on request from the corresponding author S.C. The data are not publicly available because of ethical restrictions that protect patients’ privacy and consent.

## Supplementary Materials

The following supporting information can be downloaded at: https://www.mdpi.com/article/10.3390/brainsci14111093/s1. Figure S1. Histograms of baseline demographic characteristics.

## Abbreviations

LMM	Low muscle mass
IRB	Institutional review board
HbA1c	Glycosylated hemoglobin
LDL	Low-density lipoprotein (cholesterol)
HDL	High-density lipoprotein (cholesterol)
Tg	Triglycerides
BMI	Body-mass index
BIA	Bioelectrical impedance analysis
SMI	Skeletal Muscle Mass Index
CESD	Center for Epidemiological Studies Depression (scale)
ANOVA	One-way analysis of variance
ANCOVA	Analysis of covariance
Sbp	Systolic blood pressure
OR	Odds ratio
HPA	Hypothalamus pituitary axis
HPG	Hypothalamus pituitary gonadal axis
