# Supplementary figures and images for: Sex-Specific Association of Low Muscle Mass with Depression Status in Asymptomatic Adults: A Population-Based Study
Source: Brain Sci. 2024 Oct 30;14(11):1093. doi: 10.3390/brainsci14111093 (PMC11591987; doi:10.3390/brainsci14111093)

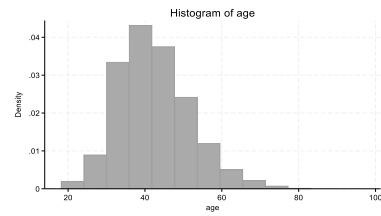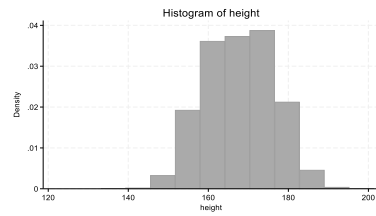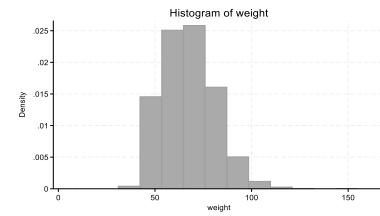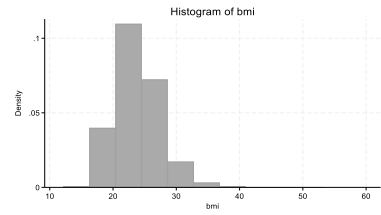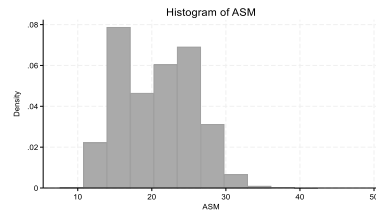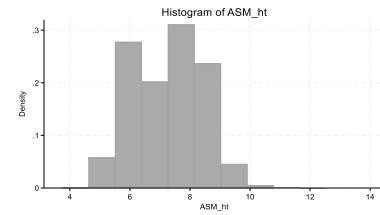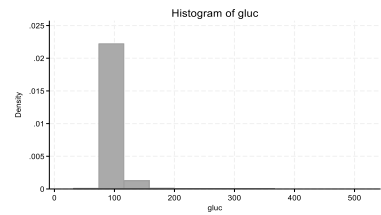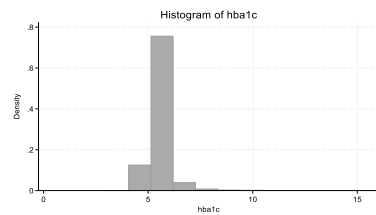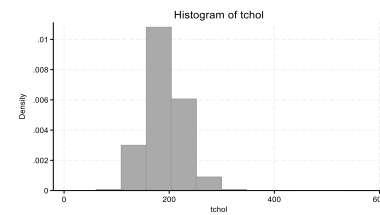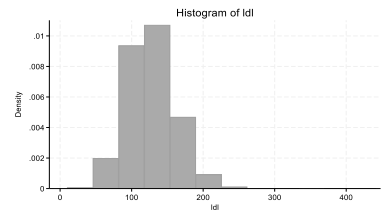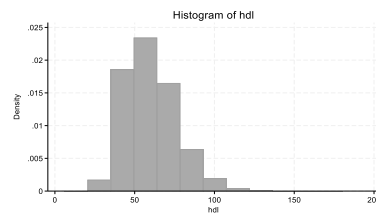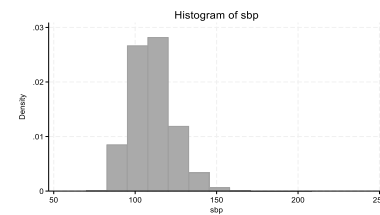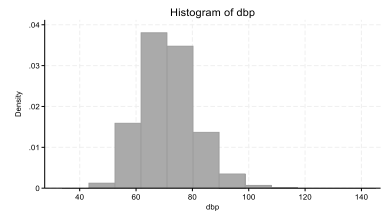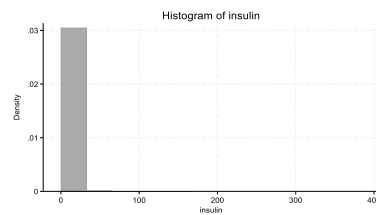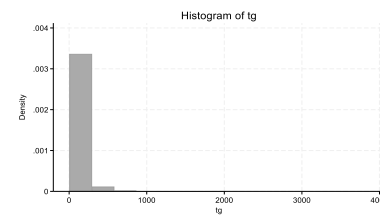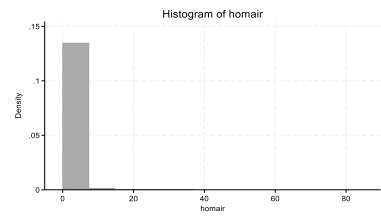

Supplement: Supplementary file 1 [file brainsci-14-01093-s001.zip › Figure S1. Histograms of baseline demographic.pdf]
